# Prognosis and aetiology of hospital-acquired pneumonia (HAP) and ventilator-associated pneumonia (VAP) in patients with active lung or lower head and neck cancer (PEPP-C): a retrospective, matched multicentre cohort study in Germany and Spain

**DOI:** 10.1007/s15010-026-02805-y

**Published:** 2026-05-04

**Authors:** Sina M. Pütz, Marie Engelhard, Sebastian M. Wingen-Heimann, David F. Brilz, Brune Akrich, Heike Kohl, Annika Y. Classen, Carlota Gudiol, Enric Sastre-Escola, Franziska C. Trudzinski, Arturo Olivares Rivera, Eva Lücke, Tadeusz Marczewski, Ayham Daher, Stephan Eisenmann, Jacqueline Schmitz, Anke Reinacher-Schick, Julia von Tresckow, Rebekka Mispelbaum, Tessa Hattenhauer, J. Janne Vehreschild

**Affiliations:** 1 Department I of Internal Medicine, Division of Infectious Diseases, Faculty of Medicine and University Hospital Cologne, University of Cologne, Kerpener Straße 62, 50937 Cologne, Germany; 2 Department 1 of Internal Medicine, Faculty of Medicine and University Hospital Cologne, University of Cologne, Cologne, Germany; 3 Faculty of Medicine, Institute for Digital Medicine and Clinical Data Science, Goethe University Frankfurt, Frankfurt, Germany; 4 Institute of Translational Research, Cologne Excellence Cluster on Cellular Stress Responses in Aging-Associated Diseases (CECAD), University Hospital Cologne, Faculty of Medicine, University of Cologne, Cologne, Germany; 5 MSD France, Puteaux, France; 6 MSD Sharp & Dohme GmbH Germany, Munich, Germany; 7 Infectious Diseases Department, Bellvitge University Hospital, B, Bellvitge Biomedical Research Institute (IDIBELL), University of Barcelona, L’Hospitalet de Llobregat, Barcelona, Spain; 8 Centro de Investigación Biomédica en Red de Enfermedades Infecciosas (CIBERINFEC), Instituto de Salud Carlos III, Madrid, Spain; 9 Infectious Diseases Unit, Catalan Institute of Oncology (ICO), Duran i Reynals Hospital, IDIBELL, Barcelona, Spain; 10 Department of Pneumology and Critical Care Medicine, Thoraxklinik, University of Heidelberg, Heidelberg, Germany; 11 Translational Lung Research Center (TLRC-H), German Center for Lung Research (DZL), Heidelberg, Germany; 12 Department of Pneumology, Otto-von-Guericke-University, Magdeburg, Germany; 13 Department of Pneumology and Intensive Care Medicine, University Hospital RWTH Aachen, Aachen, Germany; 14 Department of Internal Medicine I, University Hospital Halle (Saale), Halle, Germany; 15 Department of Hematology and Oncology with Palliative Care, Katholisches Klinikum, Ruhr University Bochum, Bochum, Germany; 16 Medical Clinic III for Oncology, Hematology, Immune-Oncology and Rheumatology, University Hospital Bonn (UKB), Bonn, Germany; 17 Center for Integrated Oncology Aachen/Bonn/Cologne/Düsseldorf (CIO-ABCD), Bonn, Germany

**Keywords:** Hospital acquired pneumonia, Ventilator associated pneumonia, Lung cancer, Lower head and neck cancer, Real-world data

## Abstract

**Purpose:**

Patients with active lung or lower head and neck cancer (HNC) are at increased risk of infection, particularly hospital-acquired pneumonia (HAP), ventilated HAP (vHAP) and ventilator-associated pneumonia (VAP), due to immunosuppression and treatment-related side effects. These infections are associated with increased mortality, prolonged hospital stays and interruptions in cancer therapy. Despite their clinical relevance, data on epidemiology, risk factors, treatment, and outcomes in this population remain limited.

**Methods:**

We conducted a retrospective, observational, multicentre matched cohort study at eight tertiary care centres in Germany and Spain. Patients with lung or lower HNC who received anti-cancer treatment between January 2018 and May 2022 and were hospitalised for at least 48 h were included. Patients with HAP/vHAP/VAP (case group) were matched 1:1 to those without pneumonia (control group) and followed for 90 days.

**Results:**

A total of 256 patients were included, with median ages of 65 (cases) and 64 years (controls). Most patients had lung cancer (94%, *n* = 240), predominantly in stages III-IV (81%, *n* = 207). In the case group, patients had mostly HAP (88%, *n* = 113), diagnosed in median 7 days after hospital admission. Common antibiotics given included penicillin derivatives with β-lactamase inhibitors, cephalosporins and carbapenems. Patients with HAP/vHAP/VAP had significantly higher mortality and longer hospital stays (*p* < 0.001). Cox regression identified HAP/vHAP/VAP and dysphagia as independent predictors of mortality.

**Conclusion:**

HAP/vHAP/VAP imposes a significant clinical burden in patients with lung or lower HNC. Early prevention, timely diagnosis, and close monitoring of high-risk patients are essential to improve outcomes.

**Supplementary Information:**

The online version contains supplementary material available at 10.1007/s15010-026-02805-y.

## Introduction

Patients with active malignancies undergoing anti-cancer therapy are at markedly increased risk of infectious complications due to treatment- and disease-related immunosuppression [1]. Pneumonia represents one of the most common and clinically significant infections [2–4], with particularly high rates of hospital-acquired pneumonia (HAP) and ventilator-associated pneumonia (VAP) observed among patients with active lung or lower head and neck cancers (HNC) [5–8].

Lung cancer remains the leading cause of cancer-related mortality [9], representing a significant burden on healthcare systems. In addition to the severity of the underlying diseases, HAP and VAP are life-threatening comorbidities that significantly increase mortality. A recent study reported significantly higher in-hospital mortality and prolonged hospital stays among patients with non–small cell lung cancer (NSCLC) who developed pneumonia, compared to patients without lung cancer [10]. Aspiration pneumonia has also been described as a frequent complication of chemotherapy (CT) and radiotherapy (RT) in patients with HNC, occurring in up to 25% of cases within 28 days of treatment initiation [11]. CT and RT further increase infection risk through side effects such as dysphagia and hematologic toxicity. While postoperative pneumonia occurs in approximately 3–4% of patients following major cancer surgery [12], its incidence is substantially higher among patients with lung and HNC [5, 13–15]. Liu et al. identified an incidence of VAP of 19.6% in patients who required more than 48 h of mechanical ventilation after major oncological surgery for HNC [5]. Another study showed that lung cancer was the most common cancer type in pneumonia episodes among patients with solid tumours [6]. Furthermore, pneumonia and complications related to anti-cancer treatment are leading causes of intensive care unit (ICU) admission in lung cancer patients [16], with those suffering from ventilated HAP (vHAP) experiencing the highest mortality during the course of the disease [17].

Several risk factors for pneumonia have been described in patients with lung and lower HNC undergoing active anti-cancer therapy, including poor oral hygiene, advanced nodal diseases, hypoalbuminemia prior to treatment, inpatient status, advanced age, current smoking, chronic obstructive pulmonary disease (COPD), a higher Simplified Acute Physiology Score system (SAPS) II upon admission, advanced stage (stage IV) lung cancer, and neutropenia [5, 11, 18].

The development of HAP/vHAP/VAP often disrupts anti-cancer treatment by causing treatment delays or even discontinuations. Furthermore, there is evidence demonstrating that exposure to broad-spectrum antibiotics, especially within a window of 30 days before immune checkpoint inhibitor (ICI) initiation, is associated with significantly shorter overall survival (OS) and progression-free survival (PFS) in patients with NSCLC and other malignancies compared to patients without antibiotic exposure [19, 20]. Consequently, there is an urgent need for effective patient management standards including optimized anti-infective treatment strategies to improve patient outcomes.

Evidence regarding the current epidemiology, risk factors, anti-infective treatment strategies, and clinical outcomes of HAP/vHAP/VAP in patients with active lung or lower HNC remains limited. Although it is assumed that HAP/vHAP/VAP and their management have a significant impact on oncological treatment and outcomes, robust clinical evidence is scarce. To address this gap, we conducted a multicentre, international, real-world study to analyse the risk factors and treatment strategies for HAP/vHAP/VAP, as well as their impact on clinical and oncological outcomes in patients with lung and lower HNC.

## Methods

### Study design and study population

We conducted a retrospective, observational, multicentre matched cohort study at eight tertiary care centres in Germany (*n* = 7) and Spain (*n* = 1). Adult patients with lung or lower HNC who received anti-cancer treatment between January 2018 and May 2022 were included. Patients had a minimum hospital stay of 48 h and were followed up for 90 days. Cases were defined as patients with HAP, vHAP or VAP, while controls had no documented HAP/vHAP/VAP. Matching was performed in a 1:1 ratio within each study site using a nearest neighbour approach based on the following criteria, in descending order of priority: sex, age at admission (+/- 15 years), reason for admission (a) surgery, b) oncological treatment (including complications of treatment or tumour progression), c) infection, d) other), type of cancer (lung cancer vs. lower HNC), cancer stages (I-IV) according to the Union for International Cancer Control (UICC), season of admission (autumn-winter vs. spring-summer). Post-matching covariate balancing was assessed using standardized mean differences (SMDs), with values < 0.1 indicating acceptable balance.

### Definitions

For the purpose of this study, we defined “lower HNC” as laryngeal or hypopharyngeal cancer. The presence of HAP/vHAP/VAP in the case group was identified according to the Johanson criteria [21, 22], defined as follows: new, persistent or progressive infiltrate of the lung diagnosed by thorax imaging (x-ray, computed tomography (CT)-scan) AND at least two criteria out of the following: (i) Fever > 38.3 °C (ii) Leukocytes > 10,000/µl or < 4,000/µl AND/OR (iii) Purulent secretion of the tracheobronchial system OR as an alternative for ICU patients with suspicion of VAP: > 6 points in the Clinical Pulmonary Infection Score (CPIS). HAP was defined as pneumonia developing ≥ 48 h after hospital admission. vHAP refers to HAP patients requiring subsequent endotracheal intubation, whereas VAP develops ≥ 48 h after endotracheal intubation [23]. HAP/vHAP/VAP definition also included patients who had been hospitalised within the preceding three months and for whom the treating physician ruled out community-acquired pneumonia. The first hospitalisation during the study period with a documented episode of HAP/vHAP/VAP in the case group was classified as “target hospitalisation”. The target hospital stay in the control group was defined based on the predefined matching criteria.

Oncological outcomes were assessed according to the Response Evaluation Criteria in Solid Tumours (RECIST), which evaluate treatment effectiveness by categorizing tumour response based on measurable changes in tumour size [24]. Multi-drug resistant (MDR) bacteria were defined according to the criteria proposed by Magiorakos et al. [25]. Isolates were classified as MDR if they were non-susceptible to at least one agent in three or more antimicrobial categories. In the current study, we assessed bacteria that were resistant to three or all of the following four antibiotic classes: penicillin derivatives, cephalosporins (3rd & 4th generation), carbapenems, fluoroquinolones.

### Study objectives

The primary objective of this study was to describe the clinical outcomes, defined as 28-day survival in patients with active lung or lower HNC who developed HAP/vHAP/VAP compared to those who did not. Secondary objectives included the assessment of 90-day survival, the incidence of HAP/vHAP versus VAP, identification of risk factors for HAP/vHAP/VAP, length of hospital stay (LOS), anti-infective treatment strategies, and pathogen distribution.

### Data collection and management

Patient data was collected from inpatient electronic medical records and anonymously documented into a web-based electronic clinical report form (eCRF), accessible via www.clinicalsurveys.net. Under the supervision of experienced internal medicine specialists, medical documentation specialists performed data collection at the participating study sites. The leading study site University Hospital Cologne provided the eCRF and handled the data monitoring and query management. To maintain patient anonymity, the timing of all events was expressed relative to the date of hospital admission, with admission defined as day 0.

### Statistical analysis

RStudio version 4.5.0 (from 2025-04-11) was used for data analysis. Demographic factors and baseline characteristics between HAP/vHAP/VAP patients and the control group were compared using Mann-Whitney U test, Student’s t-test, and Pearson’s chi-squared test as appropriate based on type and distribution of the covariate, applying a significance level of *p* < 0.05. Population characteristics were reported as frequency distribution and percentages, medians plus interquartile ranges (IQR), or means plus 95% confidence intervals (CIs), as appropriate. The 28- and 90-day survival rates were analysed via log rank-test. To determine the effect of residual confounders, Cox regression models were calculated with the time to HAP/vHAP/VAP and time to ventilation as independent variables. Kaplan-Meier plots were used for analysis of time from admission and duration of initial hospital stay to death in the case and control group. Baseline characteristics with *p* ≤ 0.10 in univariable analysis, together with clinically relevant covariates, were included in the univariable screening. All clinically relevant covariates were entered into the initial multivariable model. Variance inflation factors (VIFs) were calculated to assess multicollinearity; a VIF below 5 was considered acceptable, indicating no relevant collinearity among predictors. For the final model, backward elimination guided by the Akaike Information Criterion (AIC) was applied. Logistic regression was used for binary outcomes, and Cox proportional hazards regression for time-to-event outcomes. Consistent with the real-world design of this study, we performed complete case analyses, excluding patients with missing values at each analysis step when the missing data was completely at random.

## Results

### Study population

We analysed a total of 256 patients with lung or lower HNC, including 128 patients with (HAP/vHAP/VAP group) and 128 without HAP/vHAP/VAP (control group). Baseline characteristics are shown in Table 1. Post-match covariate balance showed that all key baseline characteristics and matching variables achieved an SMD < 0.1, except for antibacterial treatment within six months prior to the index hospitalisation, indicating only minimal residual imbalance between groups (Figure S1).

**Table 1:** Patient characteristics of the study population (*n* = 256)

	Case group (*n* = 128)	Control group (*n* = 128)	*p* value	
Female sex; *n* (%)	34 (27)	32 (25)	0.775^a^	
Age in years; median (range)	65 (34–84)	64 (47–84)	0.554^b^	
Body mass index; n (%)	124 (97)	125 (98)	0.158^a^	
< 18.5	12 (10)	2 (2)		
18.5–24.9	56 (45)	55 (44)		
25.0–29.9	39 (31)	43 (34)		
> 30.0	15 (12)	21 (17)		
Underlying disease				
Lung cancer; n (%)	120 (94)	120 (94)	0.857^a^	
NSCLC	66 (55)	69 (58)		
SCLC	24 (20)	25 (21)		
SCC	25 (21)	24 (20)		
Other	4 (3)	2 (2)		
Lower HNC; n (%)	8 (6)	8 (6)	1.000^a^	
Larynx (SCC)	4 (50)	4 (50)		
Hypopharynx (SCC)	4 (50)	4 (50)		
Cancer stages according to UICC; n (%)			0.863^a^	
I	11 (9)	11 (9)		
II	15 (12)	11 (9)		
III	35 (27)	36 (28)		
IV	66 (52)	70 (55)		
Comorbidities at day 0^§1^; n (%)				
COPD	53 (41)	41 (32)	0.120^a^	
Diabetes mellitus	28 (22)	27 (21)	0.879^a^	
Peripheral vascular disease	11 (9)	19 (15)	0.120^a^	
Cerebrovascular accident/TIA	11 (9)	13 (10)	0.668^a^	
Liver disease	9 (7)	10 (8)	0.812^a^	
Congestive heart failure	8 (6)	9 (7)	0.802^a^	
Chronic kidney disease	7 (6)	7 (6)	1.000^a^	
Other	24 (19)	10 (8)	0.010^a^*	
Any of the comorbidities	91 (71)	85 (66)	0.419^a^	
Risk factors for HAP/VAP^§^; n (%)	114 (89)	110 (86)	0.450^a^	
Smoking	107 (84)	107 (84)	0.891^a^	
Acute or chronic renal failure	19 (15)	15 (12)	0.747^a^	
Dysphagia	20 (16)	9 (7)	0.018^a^*	
Neurological disease	14 (11)	12 (9)	0.758^a^	
Immobilization	6 (5)	2 (2)	0.160^c^	
Nasogastric tube	6 (5)	1 (1)	0.169^c^	
Antibacterial treatment^§+^; n (%)	43 (34)	27 (21)	0.025^a^*	
Penicillin derivatives	26 (60)	12 (44)	0.017^a^*	
Fluoroquinolones	9 (21)	6 (22)	0.270^a^	
Cephalosporins	5 (12)	6 (22)	0.416^a^	
Macrolides	4 (9)	5 (19)	0.463^a^	
Other	9 (21)	8 (30)	0.732^a^	
MDR colonisation^+^; n (%)	10 (8)	6 (5)	0.302^a^	
MDRGN bacteria^2^	6 (60)	5 (83)	0.758^a^	
VRE	3 (30)	1 (17)	0.622^c^	
MRSA	1 (10)	0 (0)	1.000^c^	
MDRGN bacteria^3^	0 (0)	0 (0)	-	

COPD, chronic obstructive pulmonary disease; HNC, head and neck cancer; MDR, multidrug-resistant; MDRGN, multidrug-resistant Gram-negative; MRSA, methicillin-resistant *Staphylococcus aureus*; NSCLC, non-small cell lung cancer; SCC, squamous cell carcinoma; SCLC, small cell lung cancer; TIA, transient ischemic attack; UICC, Union for International Cancer Control; VRE, vancomycin-resistant *Enterococcus*

^a^ Pearson chi-square test (two-tailed)

^b^ Student t-test

^c^ Fisher´s exact test

^§^ Multiple entries per patient possible + 6 months prior to hospital admission (day 0)

^1^ As identified from documented medical history

^2^ MDRGN bacteria resistant against 3/4 of the following antibiotic classes: Penicillins, Cephalosporines (3 & 4 generation), Carbapenems, Fluoroquinolones

^3^ MDRGN bacteria resistant against 4/4 of the following antibiotic classes: Penicillins, Cephalosporines (3 & 4 generation), Carbapenems, Fluoroquinolones

* p-value < 0.05

### Risk factors for HAP/vHAP/VAP

A significantly higher rate of dysphagia was observed in the HAP/vHAP/VAP group (16%) compared to the control group (7%; *p* = 0.018). Six months prior to the target hospitalisation, significantly more patients in the HAP/vHAP/VAP group received antibacterial treatment compared to the control group (34% vs. 21%; *p* = 0.025). In univariate logistic regression, a Body Mass Index (BMI) < 18.5 (OR 3.93), the presence of dysphagia (OR 2.56) and antibacterial treatment within six months prior to hospitalisation (OR 1.89) were significantly associated with the occurrence of HAP/vHAP/VAP. Most frequently reported agents used for antibacterial treatment were penicillin derivatives in both groups (case group: *n* = 26, 20%; control group: *n* = 12, 9%), followed by fluoroquinolones (*n* = 9, 7%; *n* = 6, 5%) and cephalosporins (*n* = 5, 4%; *n* = 6, 5%). The multivariable logistic regression analysis showed that patients who were treated with antibiotics within six months prior to the target hospitalisation (OR 2.96), as well as those receiving radiotherapy (OR 3.26), had significantly higher risk of developing HAP/vHAP/VAP (Table 2).

**Table 2:** Uni- and multivariable logistic regression analyses of independent variables with impact on HAP/vHAP/VAP occurrence based on baseline characteristics and risk factors for HAP/vHAP/VAP

	Univariable logistic regression			Multivariable logistic regression^§^		
	*p*	OR	95% CI	*p*	OR	95% CI
Age	0.552	0.99	0.97–1.02	-	-	-
Female (Reference = Male)	0.775	1.09	0.62–1.90	-	-	-
BMI (Reference = 18.5–24.9)						
< 18.5	0.042*	3.93	1.17–17.93	-	-	-
25.0–29.9	0.691	0.89	0.50–1.58	-	-	-
> 30.0	0.361	0.70	0.32–1.49	-	-	-
COPD (Reference = no COPD)	0.121	1.50	0.90–2.51	-	-	-
Dysphagia (Reference = no dysphagia)	0.027*	2.56	1.14–6.15	-	-	-
Antibacterial treatment+ (Reference = no antibacterial treatment)	0.066	1.82	0.97–3.47	0.006**	2.96	1.38–6.58
MDR colonisation+ (Reference = no MDR colonisation)	0.307	1.72	0.62–5.20	-	-	-
Anti-cancer therapy completed (Reference = anti-cancer therapy ongoing)	0.797	0.93	0.52–1.65	-	-	-
Chemotherapy (Reference = no chemotherapy)	0.082	1.62	0.94–2.81	-	-	-
Immunotherapy (Reference = no immunotherapy)	0.636	0.85	0.44–1.65	-	-	-
Surgery (Reference = no surgery)	0.824	1.06	0.63–1.79	0.057	2.09	0.99–4.55
Radiotherapy (Reference = no radiotherapy)	0.091	1.70	0.92–3.18	0.024*	3.26	1.21–9.64
Antibacterials at hospital admission (Reference = no antibacterials at hospital admission)	0.821	0.93	0.47–1.81	-	-	-

* p-value < 0.05; ** p-value < 0.01

*n* = 171; Akaike information criterium: 233; Bayes information criterium: 245; deviation: 225; likelihood quotient chi-square: (*p* = 0.007)

^§^ Final model including variables retained after backward selection; + six months prior to hospitalisation

BMI, Body mass index; CI, confidence interval; COPD, chronic obstructive pulmonary disease; MDR, multi-drug resistant; OR, Odds ratio

### Clinical presentation of patients with HAP/vHAP/VAP

Among patients in the case group, 88% (*n* = 113) had HAP, 7% (*n* = 9) had vHAP, and 5% (*n* = 6) had VAP. The median time to diagnosis was 7 days after hospital admission (IQR, 4–13). Nearly all patients (98%, *n* = 125) were symptomatic with a median of three symptoms reported (IQR 2–4). The most common symptoms were dyspnoea (66%, *n* = 82), purulent sputum (62%, *n* = 78), cough (51%, *n* = 64), and fever (51%, *n* = 64).

### Overall treatment and hospitalisation

The LOS was in median ten days longer in the HAP/vHAP/VAP group compared to the control group (Table 3). A significantly higher proportion of patients in the HAP/vHAP/VAP group required treatment in an intermediate care unit (IMC) and ICU compared with controls, and HAP/vHAP/VAP patients had a significantly longer ICU LOS.

**Table 3:** Hospitalisation and treatment details

	Case group (*n* = 128)	Control group (*n* = 128)	*p* value	
Hospitalisation				
Length of initial hospitalisation (days); median (IQR)	21 (15–33)	11 (7–19)	< 0.001^a^***	
Overall length of stay (days); median (IQR)	24 (15–36)	14 (8–25)	< 0.001^a^***	
Number of hospitalisations; median (IQR)	1 (1–1)	1 (1–2)	0.011^a^*	
Rehospitalisations; n (%)	19 (15)	37 (29)	0.007^b^**	
Stay on hospital wards				
General ward; n (%)	122 (95)	128 (100)	0.029^c^*	
Total days; median (IQR)	20 (11–31)	13 (7–22)	< 0.001^a^***	
IMC; n (%)	32 (25)	14 (11)	0.003^b^**	
Total days; median (IQR)	4 (2–9)	2 (1–4)	0.120^a^	
ICU; n (%)	61 (48)	41 (32)	0.011^b^*	
Total days; median (IQR)	5 (2–10)	2 (1–4)	< 0.001^a^***	
Anti-infective therapy^§^	123 (96)	86 (67)	< 0.001^b^***	
At hospital admission; n (%)	38 (31)	31 (36)	0.362^b^	
Immunosuppressants	26 (68)	13 (42)		
Antibiotics	20 (53)	21 (68)		
Antifungals	1 (3)	4 (13)		
Antivirals	1 (3)	3 (10)		
During hospitalisation, n (%)	108 (88)	65 (76)	< 0.001^b^***	
Immunosuppressants	35 (32)	34 (52)		
Antibiotics	98 (91)	37 (57)		
Antifungals	12 (11)	5 (8)		
Antivirals	5 (5)	6 (9)		
Antibiotics^§^; n (%)	127 (99)	59 (46)	< 0.001^c^***	
Penicillin derivatives + β-lactamase inhibitor	115 (91)	37 (63)	0.046^b^*	
Duration in days; median (IQR)	8 (4–11)	8 (4–9)	0.619^a^	
Cephalosporin	35 (28)	28 (47)	< 0.001^b^***	
Duration in days; median (IQR)	5 (1–8)	2 (1–5)	0.041^a^*	
Carbapenem	33 (26)	5 (8)	0.026^b^*	
Duration in days; median (IQR)	8 (5–10)	7 (5–11)	1.000^a^	
Fluoroquinolone	19 (15)	10 (17)	0.386^b^	
Duration in days; median (IQR)	7 (6–10)	3 (3–8)	0.094^a^	
Macrolide	19 (15)	6 (10)	0.592^b^	
Duration in days; median (IQR)	4 (2–7)	6 (4–9)	0.193^a^	
Sulfonamide	5 (4)	3 (5)	0.429^c^	
Duration in days; median (IQR)	12 (4–17)	3 (2–4)	0.434^a^	
Aminoglycoside	5 (4)	2 (3)	1.000^c^	
Duration in days; median (IQR)	3 (1–10)	2 (2–2)	0.864^a^	
Polymyxin	5 (4)	1 (2)	1.000^c^	
Duration in days; median (IQR)	11 (7–34)	20	1.000^a^	
Oxazolidinone	4 (3)	0 (0)	0.576^c^	
Duration in days; median (IQR)	10 (9–15)	-	-	
Other; n (%)	10 (8)	8 (14)	0.094^b^	
Duration in days; median (IQR)	7 (3–14)	8 (7–9)	0.893^a^	
Proton pump inhibitors; n (%)	14 (11)	15 (12)	0.844^b^	
Duration in days; median (IQR)	22 (17–24)	18 (16–28)	0.585^a^	
Status of anti-cancer therapy; n (%)			0.792^b^	
Completed	30 (23)	32 (25)		
Ongoing during hospitalisation^§^	92 (72)	91 (71)		
Chemotherapy	38 (41)	25 (27)	0.065^b^	
Immunotherapy	17 (18)	19 (21)	0.855^b^	
Radiotherapy	27 (29)	16 (18)	0.116^b^	
Surgery	22 (24)	17 (19)	0.431^b^	

ICU, intensive care unit; IMC, intermediate care unit; IQR, interquartile range

^§^ Multiple entries per patient possible

^a^ Mann-Whitney-U-test

^b^ Pearson chi-square test (two-tailed)

^c^ Fisher´s exact test

* p-value < 0.05; ** p-value < 0.01; *** p-value < 0.001

Among all ICU patients, the main reasons for admission were post-operative care (26%, *n* = 27), surgery-related admission (23%, *n* = 23), respiratory failure (16%, *n* = 16), and infections (8%, *n* = 8). Oxygen therapy was administered to 77% (*n* = 98) of patients in the case group, of whom 26% (*n* = 25) required invasive mechanical ventilation. During hospitalisation, anti-cancer treatment was ongoing in 72% and 71% of patients in the HAP/vHAP/VAP and control group (*p* = 0.792) and most of these patients received chemotherapy (Table 3).

### Antibacterial treatment and microbiological findings

For the treatment of HAP/vHAP/VAP, most patients received penicillin derivatives in combination with β-lactamase inhibitors (91%) for a median duration of 8 days (IQR 4–11), cephalosporins (28%) for a median duration of 5 days (IQR 1–8), and carbapenems (26%) for a median duration of 8 days (IQR 5–10, Table 3).

MDR bacteria were detected in 15% (*n* = 19) of patients with HAP/vHAP/VAP compared to 2% (*n* = 3) of controls (*p* < 0.001). Those patients were mainly treated with penicillin derivatives in combination with β-lactamase inhibitors (53%, *n* = 10), while 11% (*n* = 2) and 5% (*n* = 1) received cephalosporins and carbapenems. In the HAP/vHAP/VAP group, multidrug-resistant Gram-negative (MDRGN) bacteria resistant to three classes of antibiotics were detected in 3% of patients (*n* = 4), and one patient harbored an MDR-*Klebsiella species* resistant to four antibiotic classes. In the control group, one patient (1%) carried an MDRGN organism. Significantly more patients in the case group had methicillin-resistant *Staphylococcus aureus* (MRSA) infections (9%, *n* = 12 vs. 2%, *n* = 2; *p* = 0.011), while vancomycin-resistant *Enterococcus* (VRE) was detected in three cases (2%).

### Overall outcome

**Fig. 1:**
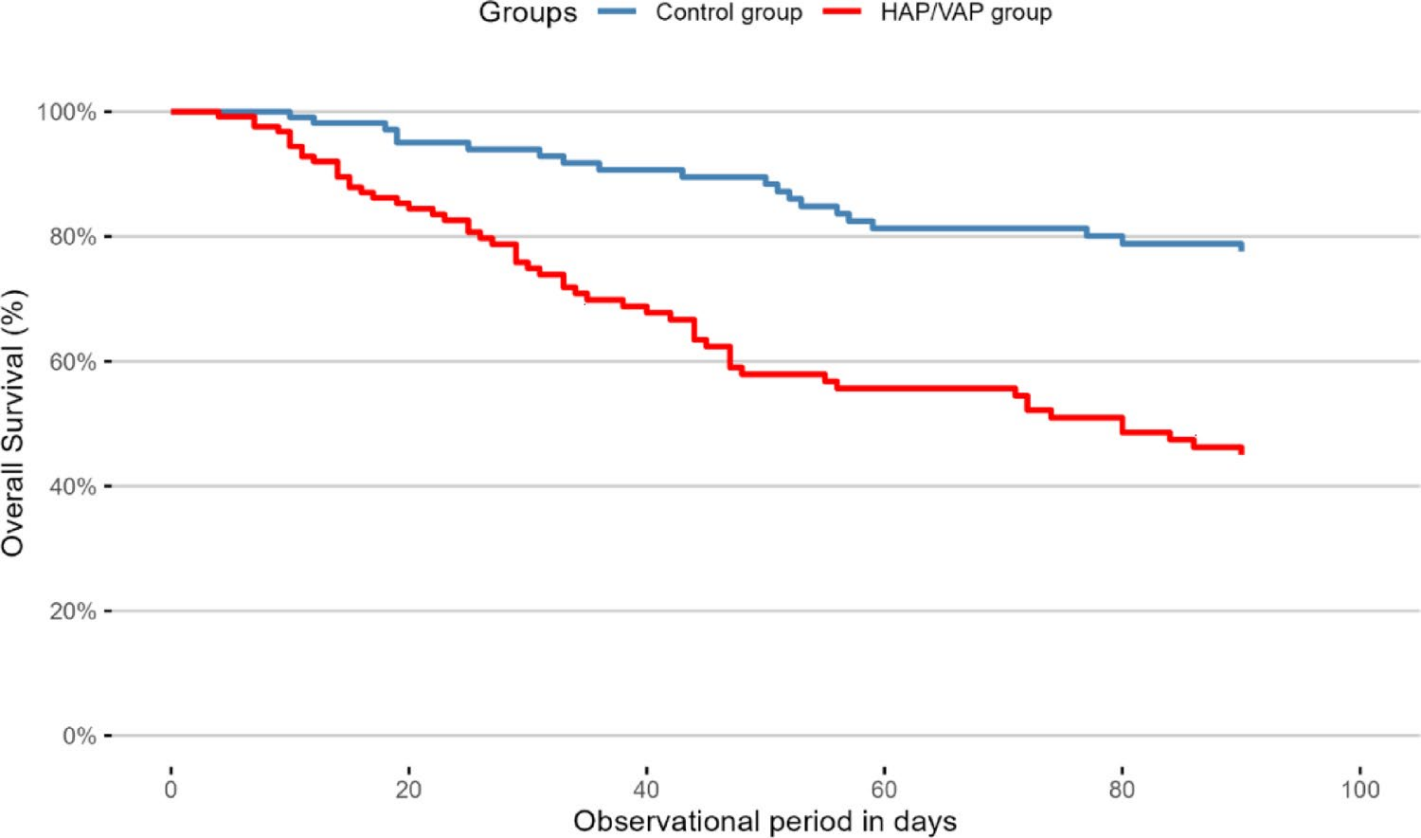
Overall survival of the observed groups (*n* = 254). Kaplan-Meier plot shows overall survival rates (in %) until day 90 following the day of hospitalisation in the control group (blue line) and the HAP/vHAP/VAP group (red line, HR = 3.30, 95% CI = 1.97–5.50, log-rank test: *p* < 0.001)

Mortality rates in the HAP/vHAP/VAP group were significantly higher compared to those of the control group at the 28- and 90- day follow up (28 days: 30%, *n* = 38 vs. 5%, *n* = 6, *p* < 0.001; 90 days: 44%, *n* = 56 vs. 16%, *n* = 20, *p* < 0.001). Kaplan–Meier analysis demonstrated significantly higher survival in the control group compared to the HAP/vHAP/VAP group at 90 days (Fig. 1). Accordingly, patients with HAP/vHAP/VAP had a significantly increased risk of death at both time points (28 days: HR 6.40; 95% CI 2.70–15.14; *p* < 0.001; 90 days: HR 3.30; 95% CI 1.97–5.50; *p* < 0.001).

Mortality differed by pneumonia subtype: at 28 days, mortality was highest in vHAP (67%, *n* = 6), followed by HAP (27%, *n* = 31) and VAP (17%, *n* = 1); at 90 days, mortality was highest in VAP (83%, *n* = 5), followed by vHAP (67%, *n* = 6) and HAP (40%, *n* = 45). Pneumonia-related causes accounted for 43% (*n* = 24) of deaths, followed by progression of the underlying malignancy (38%, *n* = 21).

At 28 days after HAP/vHAP/VAP diagnosis, 13% of patients (*n* = 16) were still receiving anti-infective therapy and 51% (*n* = 65) had been discharged. By day 90, 65% (*n* = 83) had been discharged and anti-infective treatment had been discontinued in all patients.

In the multivariable Cox regression analysis, HAP/vHAP/VAP diagnosis (HR 3.75; 95% CI 2.13–6.62; *p* < 0.001) and dysphagia (HR 2.09; 95% CI 1.13–3.85) remained independent predictors of mortality (Table 4). Time from hospital admission to HAP/vHAP/VAP diagnosis was not associated with 28- or 90-day mortality.

**Table 4:** Cox proportional hazards regression of the effect of predictors on mortality in patients with active lung or lower head and neck cancer

	Univariable cox regression			Multivariable cox regression		
	*p*	HR	95% CI	*p*	HR	95% CI
Age	0.146	1.02	0.99–1.05	-	-	-
Female (Reference = Male)	0.303	0.75	0.44–1.29	-	-	-
HAP/vHAP/VAP group (Reference = control group)	< 0.001***	3.30	1.97–5.50	< 0.001***	3.75	2.13–6.62
Lung cancer (Reference = Lower HNC)	0.013*	0.43	0.22–0.84	-	-	-
Smoking history (Reference = no smoking history)	0.244	1.82	0.66–5.01	-	-	-
Dysphagia (Reference = no dysphagia)	0.002**	2.38	1.37–4.15	0.019*	2.09	1.13–3.85
MDRGN pathogen (Reference = no MDRGN pathogen)	0.094	2.36	0.86–6.48	-	-	-
Anti-cancer therapy completed (Reference = anti-cancer therapy ongoing)	0.114	0.55	0.26–1.15	0.152	0.51	0.20–1.28
Initial hospital LOS	0.149	1.01	1.00–1.02	-	-	-
ICU stay (Reference = no ICU stay)	0.777	1.07	0.67–1.69	-	-	-

* p-value < 0.05; ** p-value < 0.01; *** p-value < 0.001

*n* = 216; likelihood ratio test: (χ² = 33.31, df = 3, *p* < 0.001)

CI, confidence interval; HNC, head and neck cancer; HR, Hazard ratio; ICU, intensive care unit; LOS, length of stay; MDRGN bacteria resistant against 3/4 or 4/4 of the following antibiotic classes: Penicillins, Cephalosporines (3 & 4 generation), Carbapenems, Fluoroquinolones

When stratified by cancer type, patients with lower HNC exhibited higher early mortality, with all deaths occurring within the first four weeks, whereas lung cancer patients showed sustained excess mortality at both time points (Table S1). In lung cancer patients, HAP/vHAP/VAP was associated with a markedly increased risk of death at 28 days (HR 3.75; 95% CI 2.11–6.68; *p* < 0.001) and 90 days (HR 4.02; 95% CI 2.26–7.17; *p* < 0.001). In contrast, mortality risk did not differ significantly between groups among patients with lower HNC at either time point (28 days: HR 1.10, 95% CI 0.32–3.79, *p* = 0.884; 90 days: HR 1.27, 95% CI 0.36–4.41, *p* = 0.711).

### Oncological outcome

At the end of the 90-day observational period, 79% (*n* = 189) of patients with lung cancer had stage III and IV disease. According to RECIST, 16% (*n* = 38) showed progressive disease, 9% (*n* = 22) had stable disease, and 11% (*n* = 26) demonstrated a partial or complete response. Among lower HNC patients, 88% (*n* = 14) had stage III and IV disease. One third presented with progressive disease (*n* = 31%, *n* = 5), 13% (*n* = 2) had stable disease and 6% (*n* = 1) achieved a complete response.

Comparison of RECIST outcomes between groups revealed overall comparable results. While complete and partial responses were more frequently observed in the control group (14%, *n* = 18 vs. 7%, *n* = 9), stable and progressive disease rates were similar (27%, *n* = 35 vs. 25%, *n* = 32, *p* = 0.202). Changes in tumour burden were unknown in approximately half of patients across both cancer types. Additional reasons for non-classifiable RECIST outcomes included prior surgical resection in 10% of patients (*n* = 13) in both groups.

When stratified by cancer type, oncological outcomes were poorer in patients with lower HNC, with no partial or complete responses and a high proportion of progressive disease in both groups at the end of follow-up (Table S1).

### Patients with vHAP

All patients with vHAP had lung cancer (stage III and IV: 89%, *n* = 8), with diabetes mellitus (67%, *n* = 6) and COPD (33%, *n* = 3) as common comorbidities. Most patients were current or former smokers (89%, *n* = 8, median 40 pack-years), and more than half had acute or chronic renal failure (56%, *n* = 5).

The median time to vHAP diagnosis was 15 days after hospital admission (IQR 3–25), occurring six or eight days later than the median diagnosis time among patients with VAP or HAP. Patients with vHAP had a substantially longer LOS compared to those with HAP (median 34 days vs. 20 days), including a median ICU stay of 10 days (IQR 7–23), primarily due to respiratory failure (33%, *n* = 3) or surgical indications (22%, *n* = 2). Additional details regarding hospitalisation, anti-infective therapy, and anti-cancer treatment across pneumonia types are provided in Table S2.

Of the vHAP patients who died during the 90-day follow-up period, all deaths occurred by day 28 (67%, *n* = 6). According to RECIST, 22% (*n* = 2) and 11% (*n* = 1) of patients showed stable disease and partial response, whereas in one patient, the disease was progressive.

## Discussion

In our study, we retrospectively analysed the influence of HAP/vHAP/VAP on the clinical and oncological outcome of 256 patients with active lung cancer or lower HNC. As primary endpoint, we found a significantly higher 28-day mortality in the HAP/vHAP/VAP group compared to the control group without HAP/vHAP/VAP. At the end of the 90-day observational period, mortality rates were significantly higher in the case group, with a risk of death being 3.3-times higher compared to controls. Our results highlight the poor outcome of patients with lower HNC or lung cancer when developing HAP/vHAP/VAP. Earlier epidemiological studies demonstrated high rates of HAP and VAP in this patient population underlining the burden of this disease [5–8].

In the study from Liu et al., patients with head and lower HNC developing VAP had mortality rates in ICU of 17% [5]. Similarly, a 30-day mortality rate of 28% has been reported in lung cancer patients with pneumonia [6], which is consistent with the 28-day mortality rate of 30% observed in patients with HAP/vHAP/VAP in our cohort. When mortality was analysed by pneumonia type, patients with vHAP exhibited the highest mortality, followed by HAP and VAP. A similar trend was observed in the study from Talbot et al. who analysed seven datasets of patients with HAP/vHAP/VAP from the United States [26]. However, they reported lower mortality rates in the vHAP (28%) and HAP groups (18%), and similar rates in VAP patients (15%).

The overall mortality rate in our HAP/vHAP/VAP cohort was 44%, which is notably higher than in previously published studies. This difference may, at least in part, be explained by the longer follow-up period of 90 days. Although HAP/vHAP/VAP was the most common cause of death (43%), a substantial proportion of patients (38%) died from progression of the underlying malignancy and might have survived the infectious episode. Nevertheless, comparison with a matched control cohort without HAP/vHAP/VAP demonstrated significantly higher mortality among patients with HAP/vHAP/VAP, underscoring the negative impact of the infection on overall outcomes in patients with lung or lower HNC.

These findings are consistent with previous reports. Kao et al. showed that 2-, 5-, and 10-year OS rates in patients with head and neck squamous cell carcinoma receiving concurrent chemoradiotherapy differed significantly between those who developed pneumonia and those who did not [27]. Similarly, lung cancer patients with pneumonia have been shown to experience significantly worse OS compared with non-pneumonia controls [18]. In addition, a higher in-hospital mortality has been reported among patients with NSCLC who developed pneumonia compared with patients without lung cancer [10].

Fernandez-Cruz et al. reported an ICU admission rate of 13% among patients with non-ventilator associated pneumonia and solid tumours, which is substantially lower than the 48% ICU admission rate observed in our study [6]. In addition, mechanical ventilation was required more frequently in our cohort (20% vs. 2%), while non-invasive ventilation was used less often (7% vs. 11%), further suggesting that patients in our cohort were more critically ill. However, it should be taken into account that 10% (*n* = 13) and 11% (*n* = 14) of patients in the case and control groups were admitted to the ICU for post-operative reasons rather than pneumonia-related complications. Consistent with this greater disease severity, ICU LOS was significantly longer in patients with HAP/vHAP/VAP compared with controls, highlighting the substantial health economic burden associated with HAP/vHAP/VAP in this population [28]. Further health economic analyses are planned outside the scope of the present study. Despite the markedly worse overall clinical outcomes, we did not observe significant differences in oncological disease status between the case and control groups. This finding should be interpreted with caution, as information on cancer disease status was missing for approximately half of the patients. Previous studies have suggested that infection-related inflammatory burden and treatment interruptions may indirectly contribute to cancer progression and poorer oncological outcomes [10, 29, 30].

Regarding antimicrobial therapy, 91% of our patients received penicillin derivatives plus β-lactamase inhibitors. In contrast, Fernandez-Cruz et al. reported piperacillin-tazobactam use in only 23% of patients [6]. Nevertheless, comparable prescribing patterns were observed for other agents, with meropenem used in 28% of patients (vs. 26% carbapenem use in our cohort) and levofloxacin in 23% (vs. 15% fluoroquinolone use in our cohort).

Current European guidelines for the management of HAP/VAP recommend initial empiric therapy with a single agent active against Gram-negatives including *P. aeruginosa* – such as imipenem, meropenem, cefepime, piperacillin/tazobactam, levofloxacin or ceftazidime – for high-risk patients without septic shock [31]. The predominance of penicillin derivates plus β-lactamase inhibitor empiric therapy in our study likely reflects the global trend towards reducing cephalosporin use for concerns of Clostridium difficile infection or colonization with ESBL-producing Enterobacterales. For high-risk patients, our operational approach aligns with the mentioned guidelines, emphasizing early Gram-negative coverage (including *P. aeruginosa*) and subsequent adaptation based on cultures and clinical response. Future work should incorporate site-specific antibiograms to optimize empiric antibiotic selection in this population. In our study, the multivariable model revealed an association of prior antibiotic intake and radiotherapy with the occurrence of HAP, vHAP or VAP. Baseline characteristics already suggested this trend as previous antibacterial use was significantly more frequent in the HAP/vHAP/VAP group. These findings are clinically plausible, since prior antibacterial treatment may contribute to altered microbial flora or increased susceptibility to infection, and radiotherapy is a risk factor for infections through treatment-related immunosuppression or dysphagia. Other studies also identified prior antibiotic use, as well as mechanical ventilation, as risk factors for VAP [33, 34]. The study from Liu et al. identified advanced age, current smoking, COPD, higher SAPS II at admission as risk factors for VAP, whereas tracheostomy appeared to be protective [5].

In our study, Cox proportional hazards regression showed not only HAP/vHAP/VAP, but also dysphagia, were independent predictors of mortality, which is consistent with dysphagia being a common complication of cancer and cancer therapy and a recognized risk factor for pneumonia. Marmor et al. reported a HR for death of 1.34 (95% CI 1.28–1.35, *p* < 0.001) in lung cancer patients with dysphagia compared to those without dysphagia [35].

Our study should be interpreted in light of several limitations: First, although regression analyses were performed to identify predictors with a potential impact on patient outcomes and the occurrence of HAP/vHAP/VAP, residual confounding by factors not captured in this study cannot be excluded, which is an inherent limitation of retrospective case–control designs [36]. Nevertheless, the rigorous and granular matching process applied to the study groups likely mitigated the impact of these unmeasured confounders, as well as the pot-match balance of baseline characteristics Second, as this is a real-world study, missing data in medical records were unavoidable. This limitation was particularly relevant for oncological outcomes, for which a substantial proportion of data were missing, thereby restricting the interpretation of these results Third, our study was conducted at tertiary care centres in Germany and Spain. Geographical conditions and different treatment guidelines can influence pathogen distribution and the anti-infective prescribing practices. Although diagnostic criteria were verified against established guideline sources, inter-site variability inherent to routine clinical practice may not have been fully captured. To reduce heterogeneity, patients with HNC were excluded, and the study population was restricted to patients with lung or lower HNC, thereby minimizing the inclusion of aspiration-related pneumonia. Fourth, given the small number of vHAP/VAP cases, results for these subgroups should be interpreted with caution. Fifth, we used the Johanson criteria to include patients in the case group. These are clinical criteria for pneumonia, since a firm microbiological diagnosis of pneumonia is often clinically impossible. While this is the general standard of reporting, we cannot rule out with absolute certainty that non-infectious causes such as immune checkpoint inhibitor–related pneumonitis were also responsible for the pneumonia. Despite the mentioned limitations, a key strength of our study is its multicentre, international, matched, real-world design, which enables robust comparisons between the study groups and enhances the generalizability of the findings to routine clinical practice.

## Conclusion

Our study demonstrates that patients with lung or lower HNC who developed HAP, vHAP, or VAP experience significantly worse clinical outcomes, including prolonged hospitalisation, increased ICU admission rates, and higher mortality. The study also identified the occurrence of HAP/vHAP/VAP and dysphagia as independent predictors of mortality and highlighted the clinical relevance of tailored treatment strategies. These findings underscore the substantial burden of HAP/vHAP/VAP in this vulnerable population and emphasize the need for improved preventive and therapeutic strategies.

## Supplementary Information

Below is the link to the electronic supplementary material.

Supplementary Material 1

## Data Availability

The authors are not permitted to disclose the study data beyond the research team. However, the data can be accessed through direct request to the corresponding author, and R scripts used to analyse the data can be made available on reasonable request.
