# Supplementary material for: Prognosis and aetiology of hospital-acquired pneumonia (HAP) and ventilator-associated pneumonia (VAP) in patients with active lung or lower head and neck cancer (PEPP-C): a retrospective, matched multicentre cohort study in Germany and Spain
Source: Infection. 2026 May 4;54(4):1861–73. doi: 10.1007/s15010-026-02805-y (PMC13476287; doi:10.1007/s15010-026-02805-y)
Supplement: Supplementary file 1 [file 15010_2026_2805_MOESM1_ESM.docx]

**Supplementary Material** - Prognosis and Aetiology of Hospital-acquired Pneumonia (HAP) and Ventilator-associated Pneumonia (VAP) in Patients with Active Lung or Lower Head and Neck Cancer (PEPP-C): A retrospective, matched multicentre cohort study in Germany and Spain

**Figure S1**: Post-match covariate balance measured using standardized mean difference

**Table S1:** Clinical and oncological outcome stratified by cancer type

|  | **Lung cancer (n = 240)** | | | **Lower head and neck cancer (n = 16)** | | |
| --- | --- | --- | --- | --- | --- | --- |
|  | **Case group**  **(n = 120)** | **Control group (n = 120)** | **p value** | **Case group**  **(n = 8)** | **Control group (n = 8)** | **p value** |
| **Outcome at 28 days; n (%)** | | | | | | |
| Anti-infective treatment ongoing | 15 (13) | - | - | 1 (13) | - | - |
| Hospital discharge | 62 (52) | - | - | 3 (38) | - | - |
| Death | 33 (28) | - | - | 5 (63) | - | - |
| **Outcome at 90 days; n (%)** | | | | | | |
| Anti-infective treatment ongoing | 0 (0) | - | - | 0 (0) | - | - |
| Hospital discharge | 80 (67) |  |  | 3 (38) | - | - |
| Death | 51 (43) |  |  | 5 (63) | - | - |
| **Oncological outcome; n (%)** | | | | | | |
| Complete response/ remission | 2 (2) | 5 (4) | 0.281^a^ | 1 (13) | 0 (0) | 1.000^a^ |
| Partial response | 6 (5) | 13 (11) | 0.090^b^ | 0 (0) | 0 (0) | - |
| Stable disease | 11 (9) | 11 (9) | 0.984^b^ | 0 (0) | 2 (25) | 0.467^a^ |
| Progressive disease | 18 (15) | 20 (17) | 0.703^b^ | 3 (38) | 2 (25) | 1.000^a^ |
| Other | 18 (15) | 14 (12) | 0.463^b^ | 0 (0) | 0 (0) | - |
| Unknown | 65 (54) | 56 (47) | 0.272^b^ | 4 (50) | 4 (50) | 1.000^a^ |
| ^a^ Fisher´s exact test | | | | | | |
| ^b^ Pearson chi-square test (two-tailed) | | | | | | |

**Table S2:** Hospitalisation and treatment details of the case group stratified by type of pneumonia

|  | **vHAP (n = 9)** | **HAP (n = 113)** | **VAP (n = 6)** | **p value** |
| --- | --- | --- | --- | --- |
| **Hospitalisation** | | | | |
| Length of initial hospitalisation (days); median (IQR) | 34 (18-61) | 20 (14-30) | 61 (45-75) | <0.001^a^*** |
| Overall length of stay (days); median (IQR) | 34 (18-61) | 23 (15-33) | 61 (45-75) | <0.001^a^*** |
| Number of hospitalisations; median (IQR) | 1 (1-1) | 1 (1-1) | 1 (1-1) | 0.435^a^ |
| Rehospitalisations; n (%) | 0 (0) | 18 (16) | 1 (17) | 0.493^b^ |
| **Stay on hospital wards** | | | | |
| **General ward**; n (%) | 7 (78) | 111 (98) | 4 (67) | 0.002^b^** |
| Total days; median (IQR) | 19 (13-25) | 20 (12-31) | 15 (8-25) | 0.692^a^ |
| **IMC**; n (%) | 6 (67) | 23 (20) | 3 (50) | 0.003^b^** |
| Total days; median (IQR) | 3 (2-8) | 4 (2-7) | 10 (10-20) | 0.111^a^ |
| **ICU**; n (%) | 9 (100) | 46 (41) | 6 (100) | <0.001^b^*** |
| Total days; median (IQR) | 10 (7-23) | 4 (2-7) | 41 (28-52) | <0.001^a^*** |
| **Anti-infective therapy^§^** | 9 (100) | 108 (96) | 6 (100) | 1.000^b^ |
| **At hospital admission; n (%)** | 3 (33) | 32 (28) | 3 (50) | 0.434^b^ |
| Immunosuppressants | 2 (22) | 22 (20) | 2 (33) |  |
| Antibiotics | 1 (11) | 16 (14) | 3 (50) |  |
| Antifungals | 0 (0) | 1 (1) | 0 (0) |  |
| Antivirals | 0 (0) | 1 (1) | 0 (0) |  |
| **During hospitalisation, n (%)** | 9 (100) | 95 (84) | 4 (67) | 0.142^b^ |
| Immunosuppressants | 4 (44) | 30 (27) | 1 (17) |  |
| Antibiotics | 8 (89) | 87 (77) | 3 (50) |  |
| Antifungals | 2 (22) | 10 (9) | 0 (0) |  |
| Antivirals | 1 (11) | 3 (3) | 1 (17) |  |
| **Antibiotics^§^; n (%)** | 8 (89) | 113 (100) | 6 (100) | 0.117^b^ |
| Penicillin derivatives + β-lactamase inhibitor | 7 (32) | 102 (48) | 6 (35) | 0.468^b^ |
| Duration in days; median (IQR) | 8 (4-15) | 8 (5-9) | 9 (5-14) | 0.046^a^* |
| Cephalosporin | 1 (5) | 32 (15) | 2 (12) | 0.227^b^ |
| Duration in days; median (IQR) | 8 (na) | 4 (1-9) | 6 (na) | 0.041^a^* |
| Carbapenem | 7 (32) | 23 (11) | 3 (18) | 0.008^b^** |
| Duration in days; median (IQR) | 6 (5-7) | 8 (6-10) | 10 (9-11) | 0.702^a^ |
| Fluoroquinolone | 1 (5) | 16 (8) | 2 (12) | 0.746^b^ |
| Duration in days; median (IQR) | 1 (na) | 7 (6-8) | 33 (23-43) | 0.067^a^ |
| Macrolide | 1 (5) | 17 (8) | 1 (6) | 0.756^b^ |
| Duration in days; median (IQR) | 1 (na) | 4 (3-6) | 7 (na) | 0.786^a^ |
| Sulfonamide | 1 (5) | 4 (2) | 0 (0) | 0.634^b^ |
| Duration in days; median (IQR) | 1 (na) | 15 (10-21) | - | 0.406^a^ |
| Aminoglycoside | 1 (5) | 3 (1) | 1 (6) | 0.075^b^ |
| Duration in days; median (IQR) | 19 (na) | 1 (1-3) | 2 (na) | 0.287^a^ |
| Polymyxin | 0 (0) | 3 (1) | 2 (12) | 0.080^b^ |
| Duration in days; median (IQR) | - | 11 (9-23) | 21 (na) | 0.406^a^ |
| Oxazolidinone | 0 (0) | 4 (2) | 0 (0) | 1.000^b^ |
| Duration in days; median (IQR) | - | 10 (9-15) | - | - |
| Other; n (%) | 3 (14) | 7 (3) | 0 (0) | 0.134^b^ |
| Duration in days; median (IQR) | 4 (3-5) | 10 (5-16) | - | 0.848^a^ |
| **Proton pump inhibitors; n (%)** | 0 (0) | 13 (12) | 1 (17) | 0.490^b^ |
| Duration in days; median (IQR) | - | 21 (16-23) | 96 (na) | 0.369^a^ |
| **Status of anti-cancer therapy; n (%)** |  |  |  | 0.454^b^ |
| Completed | 1 (11) | 29 (26) | 0 (0) |  |
| Ongoing during hospitalisation^§^ | 6 (67) | 80 (71) | 6 (100) |  |
| Chemotherapy | 3 (38) | 30 (36) | 5 (84) | 0.023^b^* |
| Immunotherapy | 3 (38) | 12 (14) | 0 (0) | 0.015^b^* |
| Radiotherapy | 2 (25) | 24 (29) | 1 (17) | 0.819^b^ |
| Surgery | 3 (38) | 19 (23) | 0 (0) | 0.226^b^ |
| ICU, intensive care unit; IMC, intermediate care unit; IQR, interquartile range | | | | |
| ^§^ Multiple entries per patient possible | | | | |
| ^a^ Kruskal-Wallis rank sum test | | | | |
| ^b^ Fisher´s exact test | | | | |
| * p-value <0.05; ** p-value <0.01; *** p-value <0.001 | | | | |

**Table S3**: Overview of variables with missing values

| **Parameter** | **Missing values n (%)** | | |
| --- | --- | --- | --- |
|  | **Total (N = 256)** | **HAP/vHAP/VAP group (n = 128)** | **Control group (n = 128)** |
| Tumor classification according to RECIST | 130 (50.8) | 69 (53.9) | 61 (47.7) |
| Antibacterial treatment 6 months prior to hospitalisation | 73 (28.5) | 39 (30.5) | 34 (26.6) |
| Smoking history | 23 (9.0) | 11 (8.6) | 12 (9.4) |
| Immobilization | 21 (8.2) | 13 (10.2) | 8 (6.3) |
| Dysphagia | 14 (5.5) | 9 (7.0) | 5 (3.9) |
| Nasogastric tube | 13 (5.1) | 7 (5.5) | 6 (4.7) |
| Body Mass Index | 12 (4.7) | 6 (4.7) | 6 (4.7) |
| Anti-cancer treatment completed | 11 (4.3) | 6 (4.7) | 5 (3.9) |
| Immunosuppressive therapy | 11 (4.3) | 6 (4.7) | 5 (3.9) |
| Acute or chronic renal failure | 9 (3.5) | 5 (3.9) | 4 (3.1) |
| Immunotherapy | 9 (3.5) | 5 (3.9) | 4 (3.1) |
| Neurological disease | 8 (3.1) | 5 (3.9) | 3 (2.3) |
| Cancer stadium according to UICC at the end of the observational period | 6 (2.3) | 1 (0.8) | 5 (3.9) |
| Antibiotic therapy | 4 (1.6) | 1 (0.8) | 3 (2.3) |
| Radiotherapy | 2 (0.8) | 1 (0.8) | 1 (0.8) |
| Follow-up period | 2 (0.8) | 1 (0.8) | 1 (0.8) |
| Cancer stadium according to UICC at baseline | 1 (0.4) | 1 (0.8) | - |
| Antifungal therapy | 1 (0.4) | - | 1 (0.8) |
| Antiviral therapy | 1 (0.4) | - | 1 (0.8) |
| Chemotherapy | 1 (0.4) | - | 1 (0.8) |
| Surgery | 1 (0.4) | - | 1 (0.8) |
| UICC, Union for International Cancer Control; RECIST, Response Evaluation Criteria in Solid Tumours | | | |
